# Young adults’ perspective of global environmental risks: A study on Polish university students

**DOI:** 10.1371/journal.pone.0273393

**Published:** 2022-09-22

**Authors:** Błażej Przybylski, Emilia Janeczko, Marcin Studnicki, Ernest Bielinis, Lidia Bielinis

**Affiliations:** 1 Institute of Education, The Maria Grzegorzewska University, Warsaw, Poland; 2 Department of Forest Utilization, University of Life Sciences in Warsaw, Institute of Forest Sciences, Warsaw, Poland; 3 Department of Biometry, Institute of Agriculture, Warsaw University of Life Science, Warsaw, Poland; 4 Faculty of Agriculture and Forestry, Department of Forestry and Forest Ecology, University of Warmia and Mazury, Olsztyn, Poland; 5 Faculty of Social Science, Department of Social Pedagogy and Methodology of Educational Research, Olsztyn, Poland; Neijiang Normal University, CHINA

## Abstract

This article presents the results of a study conducted on Polish university students to verify how they assess the probability of environmental risks and their potential impact on the socio-economic situation in Poland. To this end, 703 students of public universities in Warsaw were asked to complete risk assessment questionnaires. According to the respondents, of all identified types of threats, technological risks were found to be the most probable, with the environmental ones carrying the most significant social and economic impact. Among those risks, climate change was recognised as the most probable, while environmental contamination was perceived as having the strongest potential impact on Poland. No statistically significant differences were found in the views of women and men on the probability of environmental risks and their impact on the country’s socio-economic situation. Compared with students of technical and economic faculties, students of natural sciences, education, and nursing assessed the probability of environmental risks and the strength of their potential impact in Poland as much higher. The results of the study can be used to develop a communication strategy dedicated to young people in the education of environmental risks.

## Introduction

The ultimate goal of human development is to improve the quality of human life. People expect that they will be able to live long and productive lives with good health, with access to knowledge and education, and in dignity and socially fair conditions. However, human progress does not happen independently of the environment [1]. On the contrary, it continues to stimulate the ever-increasing production, urbanisation, and consumption [2–4], while depleting the planet’s non-renewable resources and regenerative powers.

Nowadays, a tendency occurs in social sciences to identify the future with uncertainty, unpredictability and changeability. The future is a *terra incognita*: chances and threats appearing simultaneously. The credibility of all scenarios and prognoses, even those created by the most prominent experts using correct, validated methods, might be questioned when facing the variability and incalculability of the modern world. The difficulty to predict and design the future accurately does not absolve the society from the obligation to anticipate it.

In the 1990s, Ulrich Beck [5] popularised the term ‘risk society’ to describe people’s approach as the policy of stripping the Earth of its ability to live. Beck was one of the first scholars to recognise this ubiquitous risk as a product of the late modernity, with human progress and development causing an increasing number of risks that affect natural ecosystems and human health. Currently, many people warn that our technologically and economically advanced civilisation is continuing to destroy all areas important for the future existence, such as soil, water, biodiversity and climate [4], largely posing a threat to the natural environment as well as ourselves [6].

There are many classifications of global risks. For example, the Global Risks Report [7] divides them into geopolitical, technological, environmental, societal, and economic. The report identifies the following as primary risks for the natural environment: biodiversity loss and ecosystem collapse, natural disasters, failure of climate change mitigation and adaptation, man-made environmental disasters, and extreme weather events. Environmental risks are understood as any environmental hazards or processes that carry potentially negative consequences for humans and what they value [8].

The nature of contemporary risks is different from those in the past. Firstly, they are much more universal, stronger, and intense than their predecessors and show a greater tendency to accumulate, such as occurring in clusters, with considerably more severe negative effects. Secondly, their media coverage is much more ‘visual’, with every risk and/or its impact being communicated to the entire world not only in words but primarily through powerful images. Another key feature of a contemporary global risk is fear—an element that has proven to be highly effective in bringing the global community together [9] (Kleer, 2016). The lack of security, difficulty in understanding and adapting to the ever-increasing pace of life, and the constant need to make choices in life are among today’s top-priority challenges that generate stress, uncertainty for tomorrow, and isolation from society [6,10]. The rising levels of risks contribute to people’s distrust towards industry, governments, and experts even though the understanding of their impact on human health is crucial if they are to be prevented. Dramatic changes in the environment correspond to higher numbers of well-established correlations between many diseases and pollution [11]. Given the growing recognition of the risk perception and the goals of environmental justice, policymakers are increasingly inclined to promote citizens’ participation in environmental management [12].

The understanding of environmental risks and decision-making mechanisms behind their prevention has been studied also by numerous authors. According to many of them, such decisions are frequently determined by beliefs and desires (or broadly defined values) [13]. Risk perception has been shown to be influenced by a number of factors, such as gender, age, location, income, family size [11,14–20]. The perception of general risks related, for example, to climate change is based on values shared by populations [21]. In addition, political and religious values have an effect on people’s belief in climate change and its communication [22]. Blennow et al. [23] found a strong correlation between decisions acknowledging the need to adapt to climate change and the awareness of its local impact combined with the actual experience of climate change and its consequences. Braman et al. [24] argue that society’s indifference to environmental risks, particularly climate change, may be related to the general understanding of their significance and the insufficient public knowledge of climate change and its consequences.

Additionally, the latest international research across 10 countries show the depth of anxiety many young people are feeling about climate change. Nearly 60% of the young people surveyed mentioned that they felt very worried or extremaly worried. More than 45% of the questioned people said that feelings about the climate negatively affected their everyday lives. Threequarters of them expressed they throught the future was frightening. More than a half (56%) said they think humanity is doomed. Two-thirds reported feeling sad, afraid and anxious. Respondents rated governmental responses to climate change negativel and reported greater feelings of betrayal than of reassurance [25].

Although the environmental issue is apparently gaining more and more publicity, education policy in Central and Eastern European countries has not yet taken decisive steps to integrate modern environmental education concepts into the curricula of children and young people. Research on the ecological awareness of students of renowned universities—this group has not been the target group of this type of research so far—is all the more important as these young people will soon join the intellectual elite of the country and will decide on the future directions of economic development and other areas of life. They represent a generation that will have to face the devastation of the environment with much more determination than older generations did. Whether reversing the ecological catastrophe is possible will depend on the knowledge, competence, actions and sensitivity of today’s students.

This study aims to establish what Polish students think about the probability of various types of risks in Poland, including environmental risks other than those related to climate change, and learn their opinion on the potential impact of the identified risks on the socio-economic situation in Poland within the next decade. To this end, the following hypotheses were adopted:

H1. According to young adults, climate change is the type of environmental risk for which the probability levels and the predicted impact on the socio-economic situation in Poland are the highest.
H2. The respondents’ opinion on environmental risks depends on gender. The probability of environmental risks and their impact on the socio-economic situation in Poland are assessed as higher by women than men.
H3. There is a correlation between the respondents’ education profile (field of study) and their views regarding the probability of specific environmental risks and their impact on the socio-economic situation in Poland. The views represented by students of natural sciences differ fundamentally from those represented by students of other fields.

## Materials and methods

### Study participants

Using the non-probability sampling, a study sample of 703 respondents (K = 439; M = 252; no data available = 12) was selected from among students of prestigious public universities in Warsaw. All respondents were aged from 18 to 24. The average age was 20,5. It included students of six faculties representing six fields of science: 1) social sciences—Faculty of Pedagogy, the Maria Grzegorzewska University (APS); 2) economic sciences—Faculty of Finance and Accounting, Warsaw School of Economics (SGH); 3) medical sciences—Faculty of Nursing, Medical University of Warsaw (MUW); 4) agricultural sciences—Faculty of Forestry, Warsaw University of Life Sciences (SGGW); 5) technical sciences—Faculty of Automation and Robotics, Warsaw University of Technology (WUT); and 6) natural sciences—Faculty of Environmental Protection, Warsaw University of Life Sciences (SGGW). The sample size was calculated based on an alpha error of 0.05 and a power of 0.95, a minimum number of 100 respondents per faculty was required. Based on the field of study, the respondents were assigned to one of three groups: 1) care and education (faculties: Pedagogy and Nursing); 2) natural sciences (faculties: Forestry and Environmental Protection); and 3) technical and economic (faculties: Automation and Robotics, Finance and Accounting). The sample was not controlled for gender; the resulting proportion of females and males reflected the general demographic trends in the students’ groups included in the study.

### Procedure

The study was conducted in the first quarter of 2019 at the respective universities, using the auditorium method. It was performed in compliance with the ethical standards of the Polish Research Ethics Committee and the 1964 Declaration of Helsinki with its subsequent amendments. The funding institution including ethics committee accepted this research project. Prior to the survey, the students were informed about its scientific objectives and were assured of the anonymity and confidentiality of their responses. The average time to complete the questionnaire form was 13 minutes.

### Measurement

The questionnaire form was designed based on two other assessments: the Global Risks Perception Survey (GRPS) and the World Problem Questionnaire (WPQ) by Zdzisław Chlewiński [26]. The GPRS results have been published annually since 2006 (with 2020 marking the 15th edition of the survey) as ‘The Global Risks Report’ by the WEF. Our questionnaire included questions about the probability of global risks and the strength of their impact on the socio-economic situation in Poland. Similar to ‘The Global Risks Report’, the risks were grouped in five categories: economic, environmental, geopolitical, societal, and technological.

The respondents were first asked to assess the probability of the identified risks in Poland within the perspective of 10 years on a five-point Likert scale, whereby 1 denoted ‘will definitely not happen’ and 5 ‘will definitely happen’. Second, the respondents had to evaluate the strength of the impact for the identified risks also using a five-point scale, where 1 denoted ‘zero or minimum impact’ and 5 ‘catastrophic impact’. Of all 39 risks listed in the questionnaire, six were categorised as environmental: 1) environmental contamination; 2) extreme weather events (natural disasters, e.g. floods and hurricanes); 3) climate change; 4) man-made environmental disasters; 5) ecosystem collapse and biodiversity loss; and 6) exploitation of natural resources.

### Data analysis

The scores marking the probability of individual risks and the strength of their potential impact on the socio-economic situation in Poland were calculated as the sum of the respondents’ assessments in a given group of questions, with results presented as mean values on a normalised scale where the minimum value was 0 and the maximum value was 1. The scores have been averaged. The normalisation of the data allowed us to compare values ​​for scales composed of different numbers of individual scores. The results for individual scores are presented as mean values, whereby 1 denotes the lowest probability and 5 the highest probability for risk probability assessments, and 1 denotes ‘zero or minimum impact’ and 5 ‘catastrophic impact’ for impact assessments. The test score reliability was verified with Cronbach’s alpha (Table 1). The scales were used for the purpose of measuring the opinions of young people participating in survey on different types of risks. This approach to the scales of risks was adopted for purely exploratory reasons. Therefore, the evaluation of the measurement properties of the scales was limited to indicating their reliability; however, we did not measure their psychometric and diagnostic properties.

**Table 1:** Cronbach’s alpha for individual types of risks.

	Probability	Impact	
Scale	Cronbach’s alpha	Cronbach’s alpha	No. of items
Economic	0.624	0.541	5
Environmental	0.742	0.756	6
Geopolitical	0.725	0.794	7
Societal	0.818	0.818	18
Technological	0.630	0.595	3

The influence of predictors, such as the gender and the field of study on the assessment of the probability and the impact of risks; including environmental risks; was determined using the analysis of variance (ANOVA). The two-way ANOVA was applied to account for the interaction effect between gender and the field of study. Prior to the analysis, the assumption regarding the normal distribution and homogeneity of variance was verified. The homogeneity of variance was evaluated by Levene’s test, and the normal distribution of data was checked used Shapiro-Wilkinson test.

The conditions for the use of ANOVA were met for all risks. The Tukey test was used as a post-hoc test to determine significant differences in average scores for individual fields of study. All statistical analyses were performed, using R 4.4.1 software.

## Results

### Environmental vs. other types of risks

The study revealed that the respondents considered technological (0.61) and societal (0.61) risks as the most probable, followed by environmental (0.55), economic (0.51), and geopolitical (0.40) risks. In terms of their potential social and economic impact on Poland, the score for environmental risks was the highest (0.70) followed by geopolitical (0.68), economic (0.66), societal (0.65), and technological risks (0.60).

No statistically significant differences were found between females and males in the assessment of the risk probability (except for geopolitical risks) or the impact of any type of risk on the socio-economic situation in Poland (see Table 2).

**Table 2:** Results of the two-way ANOVA analysis for the probability (P) and the impact (I) for each type of risk for p < 0.05.

	Risks									
	Economic		Environmental		Geopolitical		Societal		Technological	
Effect	P	I	P	I	P	I	P	I	P	I
Gender	0.249	0.873	0.219	0.456	0.004	0.564	0.120	0.466	0.064	0.182
Field of study	0.045	0.200	0.000	0.000	0.008	0.700	0.000	0.170	0.299	0.003
Gender*field of study	0.413	0.648	0.417	0.849	0.456	0.086	0.175	0.380	0.455	0.551

### The probability of environmental risks and their potential impact on the socio-economic situation in Poland

**Fig 1:**
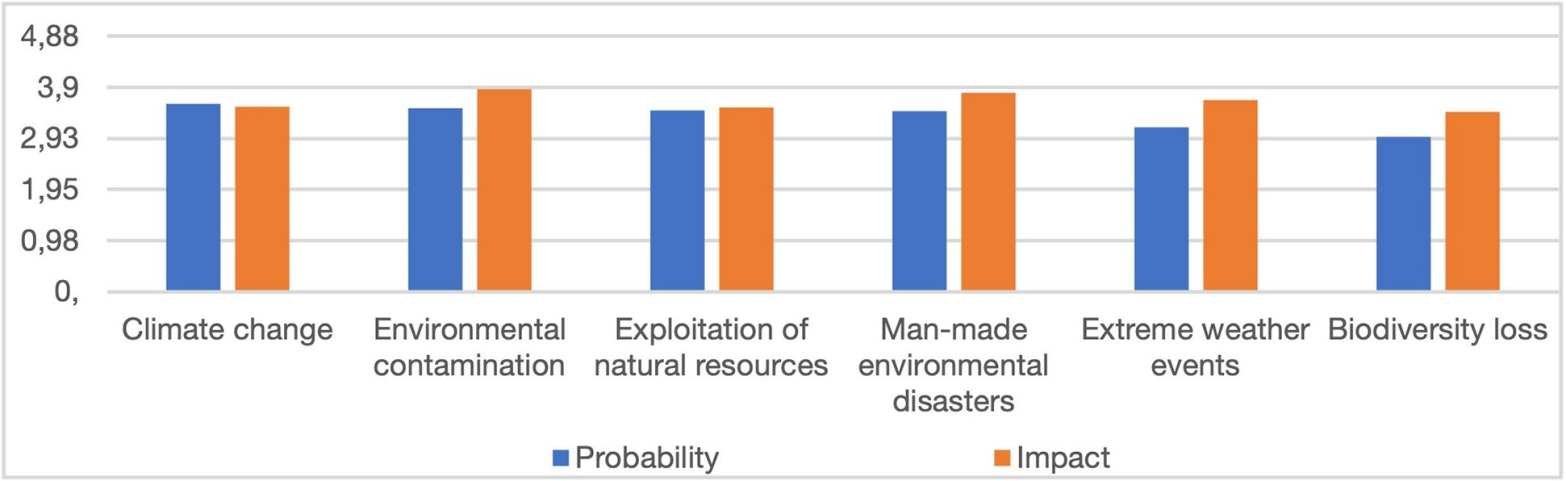
Average scores for the probability of individual environmental risks and their social and economic impact in Poland.

Of all environmental risks, the probability of the climate change (3.58) was assessed as the highest, followed by environmental contamination (3.50), exploitation of natural resources (3.46), man-made environmental disasters (3.44), extreme weather events (natural disasters, e.g. floods, hurricanes, etc.) (3.14), and ecosystem collapse and biodiversity loss (2.96). According to the respondents, the impact of the environmental contamination on the socio-economic situation in Poland was the highest (3.86), while that of biodiversity loss was found to be the lowest (3.43) (Fig 1).

**Fig 2:**
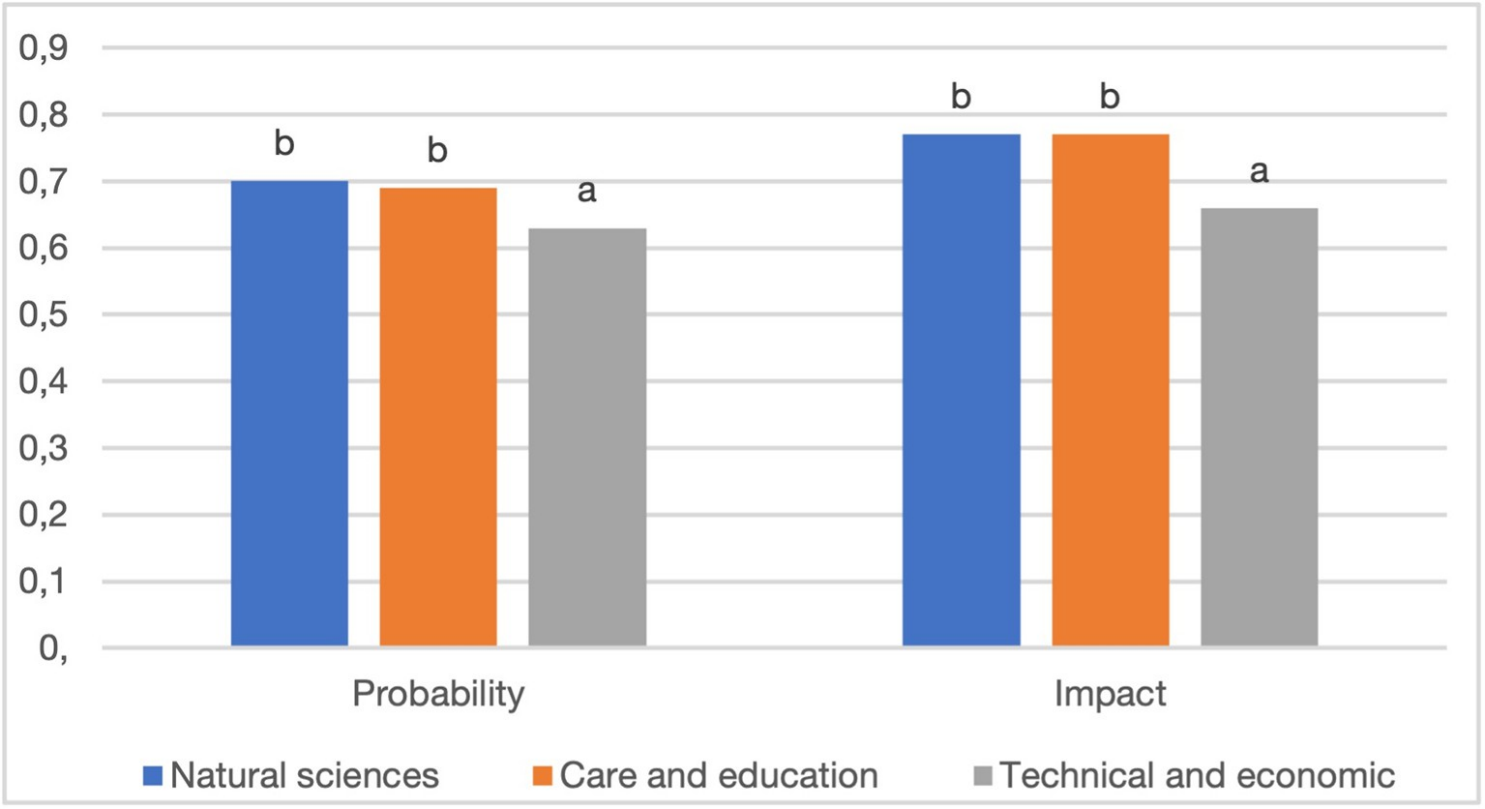
Differences in the assessment of the probability and the potential impact of environmental risks between students of different fields of study. *Note*: The letters mark significant differences between the fields of study (based on the Tukey test).

While no statistically significant differences were found between females and males in the assessment of the probability of environmental risks and their impact on the socio-economic situation in Poland, women rated both the probability and the impact as higher for each type of risk. In contrast, statistically significant differences were observed with respect to the field of study represented by the study participants. The probability of environmental risks was assessed as the highest by students of natural sciences (0.70) and the lowest by students of technical and economic faculties (0.63). Students of natural sciences, on par with students of the care and education faculties, recognised the impact of environmental risks as the most significant (0.77 in both cases), while those of the technical and economic faculties perceived it as the lowest (0.66). Consequently, students of natural sciences were the only group of students in which environmental risks ranked first both in terms of their probability and impact on Poland (Fig 2).

### The probability and the impact of individual environmental risks in terms of predictors such as gender and the field of study

**Fig 3:**
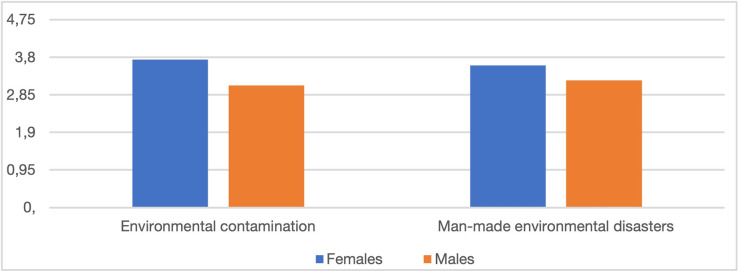
The probability of specific environmental risks according to women and men.

Males and females differed in their probability assessments of two environmental risks, environmental contamination (p = 0.0021) and man-made environmental disasters (p = 0.0360), with women assessing their probability higher than men (Fig 3). No similar relationships were found with regard to other environmental risks. Neither did we observe any gender-specific differences in the respondents’ assessment of the impact of individual environmental risks on the socio-economic situation in Poland.

Taking the field of study into account, differences were found in the respondents’ probability assessments for five out of six environmental risks identified in the questionnaire: climate change (p < 0.0001), environmental contamination (p = 0.0114), man-made environmental disasters (p = 0.0072), extreme weather events (p < 0.0001), and biodiversity loss (p = 0.0057). In terms of the impact assessment, the field of study was found not to correlate only with environmental contamination (Table 3).

**Table 3:** Results of the two-way ANOVA for probability (P) and impact (I) of individual environmental risks with p < 0.05.

Effect	Climate change		Environmental contamination		Exploitation of natural resources		Environmental disasters		Extreme weather events		Biodiversity loss	
	P	I	P	I	P	I	P	I	P	I	P	I
Gender	0.3155	0.0535	**0.0021**	0.6737	0.1581	0.1930	**0.0360**	0.0817	0.8058	0.1414	0.1223	0.9535
Field of study	**<0.0001**	**<0.0001**	**0.0114**	0.3327	0.5396	**0.0065**	**0.0072**	**<0.0001**	**<0.0001**	**<0.0001**	**0.0057**	**0.0009**
Gender* field of study	0.0661	0.5090	0.4266	0.9912	0.7636	0.6199	0.8640	0.4879	0.3111	0.7148	0.6052	0.6999

Compared with students representing care and education faculties as well as economic and technical faculties, students of natural sciences assessed four out of the six identified environmental risks as more probable—climate change (3.88), environmental contamination (3.90), exploitation of natural resources (3.60), and extreme weather events (3.51)—and only two as having a greater impact on the socio-economic situation in Poland—exploitation of natural resources (3.71) and extreme weather events (3.96). In general, students of technical and economic faculties tended to assess both the probability and the impact of environmental risks as much lower than the remaining respondents (particularly in three categories: environmental contamination, environmental disasters, and biodiversity loss; see Table 4).

**Table 4:** Post-hoc test results confirming significant differences in average scores for individual fields of study.

	Climate change		Environmental contamination		Exploitation of natural resources		Environmental disasters		Extreme weather events		Biodiversity loss	
Post-hoc test	P	I	P	I	P	I	P	I	P	I	P	I
Natural sciences	3.88b	3.16a	3.90c	3.46b	3.60c	3.71c	3.55b	3.97b	3.51c	3.96b	3.00b	3.55b
Care and education	3.54a	3.62b	3.62b	3.87c	3.35a	3.54b	3.69b	4.04b	3.11b	3.81b	3.29c	3.63b
Technical and economic	3.37a	3.90c	3.16a	3.21a	3.54b	3.34a	3.17a	3.46a	2.81a	3.29a	2.70a	3.13a

*Note*: The letters mark significant differences between the fields of study (based on the Tukey test).

The probability of climate change was recognised as higher by students of natural sciences compared with other respondents. This was also the only type of risk for which the opinion of the respondents studying care and education was similar to that represented by students of technical and economic faculties. Significantly, students of natural sciences assessed the impact of climate change on the socio-economic situation in Poland as the lowest, whereas the assessment by those studying technical and economic sciences as the highest (Table 4). The respondents’ opinions regarding the probability and the impact of environmental contamination and exploitation of natural resources varied considerably across the fields of study. The impact of either of them was rated the lowest by students of technical and economic faculties. Environmental disasters and extreme weather events also ranked the lowest by students of technical and economic faculties, both in terms of their probability and impact. In contrast, the probability levels and the impact of environmental disasters were assessed as the highest by the respondents studying care and education. Similar results for both categories (probability and impact) were recorded for biodiversity loss—the lowest among students of technical and economic sciences, and the highest among those studying care and education.

## Discussion

### H1. According to young adults, climate change is the type of environmental risk for which the probability levels and the predicted impact on the socio-economic situation in Poland are the highest

Firstly, according to the students who participated in the study, environmental risks are expected to have the greatest impact on Poland’s socio-economic situation. While climate change seems to be the most probable of them all, its potential impact has been found to be lower compared with other environmental risks. Consequently, the first hypothesis adopted in our study has not been confirmed. In general, the perception of environmental risks is strongly related to external factors, and it differs depending on the geographic location and the time of the study [27]. For example, previous studies conducted in Poland [28] highlight the importance of information on environmental risks and their consequences in the mass media, particularly on the Internet. In Poland, environmental issues have received broad media coverage in recent years. The media have a specific ability to stimulate the environmental debate and promote actions aimed at the protection of natural environment. The media coverage of environmental risks, in particular those related to climate change, has also been the result of the increasing expectations voiced ever more loudly by climate movements. In the public space (i.e. among non-specialists), climate change is being used as a synonym for environmental changes. Being part of the common knowledge all over the world, this phenomenon is currently identified as the most severe of problems [29].

### H2. The respondents’ opinion on environmental risks depends on gender. The probability of environmental risks and their impact on the socio-economic situation in Poland are assessed as higher by woman than men

Secondly, our study has found no statistically significant differences between men and women concerning their assessment of the probability and the potential impact of environmental risks. Therefore, the second hypothesis has been rejected. Similar conclusions have been presented before, for example by Slimak and Dietz [30], who discard gender as a differentiating factor in people’s perception of environmental risks, indicating that age plays a more important role in this regard. Mohai [31] also argues that there are no differences between women and men regarding their perception of environmental risks, suggesting that discrepancies in the approach to environmental issues may be better explained by ethnic origin; for example, African Americans assessed the probability of most environmental risks as higher than White Americans. In his earlier research, Mohai [32] observed that women expressed a greater concern about environmental issues; however, the differences were small. In contrast, men were found much more environmentally active. Blocker and Ecberg [33] also repudiate the popular thesis that environmental issues lie more within the interest area of women rather than men. Their study shows no significant differences between females and males in their perception of risks related to environmental pollution or natural resources; however, women taking care of young children are at the same time less willing to favour economic development at the expense of environmental protection. Furthermore, Sjoberg [34] observes that gender accounts for just over 1% of variance in risk assessments. Additionally, Kalof et al. [35] argue that gender differences in the perception of environmental risks, if any, are small.

Nevertheless, there is also ample research showing that women are more strongly convinced about the likelihood and consequences of environmental risks than men. According to Barke et al. [18], one of the most consistent conclusions from research on the risk perception is that women express greater concern about many health and environmental risks than men. Women are more sensitive to environmental issues and have more favourable environmental attitudes than men [36]. They are more interested in living in a safe environment and staying healthy, while men tend to consider the environment as a resource to be used [37].

Feingold [38] and Randler et al.[39] explain this observation with the fact that women are more anxious than men in terms of general anxiety or neuroticism. McLean and Anderson [40] show that women tend to experience greater fear and aversion towards various phenomena than men. Davidson and Freudenburg [19] believe that, due to the role of parents or caregivers assigned to them, women show more concern about the environment and care more about environmental changes. Studying the factors responsible for the perception of environmental risks, Flynn et al. [20] also observe that white women perceive these risks as much greater compared with white men. Interestingly, these differences do not seem to apply to non-white men and women, whose risk perception is quite similar. These findings suggest that socio-political factors such as power and status are strong determinants of people’s perception and acceptance of risk.

On the other hand, one can also find studies where environmental issues appear to be more important for men than women. For example, Lazo et al. [41] argue that men are more concerned about environmental risks than women. According to [42], men’s worldviews are more oriented to environmental issues. Based on Arcury et al. [43], males are more anxious about acid rain and have a slightly greater knowledge about the causes and consequences of acid rain. Additionally, other studies show a positive sign for the most of the adaptation measures, indicating a positive relationship between gender and flood risk management tools [14]. They found that men are dominate in both indoor and outdoor activities and are responsible for any kind of risk-reduction strategies.

### H3. There is a correlation between the respondents’ education profile (field of study) and their views regarding the probability of specific environmental risks and their impact on the socio-economics situation in Poland. The views represented by students of natural sciences differ fundamentally from those represented by students of other fields

Thirdly, our study has shown a relationship between the type of studies and the respondents’ assessments of the probability and impact of environmental risks on the social and economic situation in Poland, with the highest scores observed among students of natural sciences. Consequently, the third hypothesis has been confirmed. Other research also suggests a correlation between knowledge, its type and level, and the perception of risk. According to a popular thesis, education can explain differences in how people understand and perceive environmental risks [44]. On the one hand, Barke and Jenkis-Smith [44] suggest that field of research may be associated with researchers’ perception of risk. In their study, the risk related to the nuclear-waste cycle was assessed as the lowest by scientists—men specialising in physical sciences and women specialising in exact sciences. According to [30], more educated people with a higher material status are less concerned about elements of environmental risk. The ranking of risks presented in this study proved to be statistically different for non-specialists and risk experts: the former were more interested in risks of low probability and severe consequences (e.g. hazardous waste landfills, sewage, radiation), while the latter in risks of global impact such as global warming or ozone layer depletion. Moreover [34] argues that education has a weak but positive effect on individual risk assessments, [41] show that education has a positive; albeit insignificant; effect on environmental risk scores, while other studies [45] conclude that well-educated people care more about the environment than other respondents. On the other hand, [20] claim that education is inversely related to risk perception. Finally, a representative youth survey conducted in Germany [46] shows that secondary school students, graduates, and people with higher education are particularly sensitive to environmental issues and actively involved in environmental and climate protection.

Finally, to clarify our discussion, we added a table with completed thoughts from the leading scientific articles (Table 5).

**Table 5:** Included studies for a qualitative discussion.

Author	Main conclusions
[20]	These results suggest that sociopolitical factors such as power, status, alienation, and trust are strong determiners of people’s perception and acceptance of risks. White women perceived risks to be much higher than did white men
[19]	Women tend to express higher levels of concern toward technology and the environment than do men. Increased knowledge will not lead to decreased concern. Women tend to express greater concern than do men about the health and safety implications of any given level of technological risk.
[18]	Gender differences and field of research have an additive effect on risk perceptions, with women scientists and life scientists perceiving greater risks.
[17]	Flood risk perception is strongly linked to socioeconomic variables such as age, education, house ownership, family size, past flood experience, and distance from the nearest river source, as well as institutional factors such as access to credit and extreme weather forecast information
[31]	In this study, gender differences were examined along five specific dimensions or sets of environmental issues: resource conservation, nature protection, pollution, global environmental problems and neighborhood environmental problems. Women were found to express greater concern than men over most dimensions, although differences were modest.
[30]	Social-structural variables do have some influence on risk perception. The more educated and financially well-off are less concerned about the risk items.
[41]	Both experts and laypeople tend to perceive GCC (global climate change) risks to ecosystems as less avoidable and more acceptable than risks from other causes. Compared to laypeople’s perceptions, though, experts perceived GCC risks to have slightly lower impacts, be less avoidable, more acceptable, and less understandable than non-GCC risks to ecosystems. These findings may help guide efforts to communicate with laypeople about ecological risks from climate change.
[24]	Members of the public with the highest degrees of science literacy and technical reasoning capacity were not the most concerned about climate change. The result suggests that public divisions over climate change stem not from the public’s incomprehension of science but from a distinctive conflict of interest.
[27]	There is a relationship between high functional health literacy and higher perception of global health risk and trust in institutions as sources of information and protectors against environmental hazards
[14]	Gender has a positive sign for the most of the adaptation measures, indicating a positive relationship between gender and flood risk management tools. The location as an important factor in determines the choice of mitigation measures in developing countries

## Conclusions

In a world of rapid change, in a society of constant risk and uncertainty, one of the basic features is its unpredictability [47]. Studies on disaster risk are mainly concentrating on people’s perceptions of risk [17]. The risk assessment itself is certainly influenced by various factors: young people’s interests, knowledge of the issues, media reports or the current socio-political situation. Therefore, these results are only a contribution to the discussion on the level of knowledge about global risks and the potential of global education for sustainable development.

The study revealed interesting findings. We pointed out three main key findings from the study. Firstly, environmental threats in the opinion of Polish students are those with the greatest potential impact on the future of our country. Secondly, no statistically significant differences were observed between women and men in the assessment of the possibility of occurrence and potential strength of impact of the threats. Thirdly, young people studying natural sciences rated the probability and potential impact of environmental threats higher than representatives of other faculties.

The results of the study lead to the obvious conclusion that the dissemination of ecological knowledge in all fields of study educating the country’s future intellectual elite is currently one of the most important tasks. Academic curricula should be enriched with content that sensitises young people to ecological problems and builds their pro-ecological awareness. It is worth involving students of natural sciences in this process, who could act at universities as multipliers of Earth and environmental sciences, initiate scientific circles of ecologists, but also carry out ecological lobbying activities, because—despite eagerly expressed ecological declarations by decision-makers—at national, regional and local levels degradation of the natural environment still takes place (e.g. cutting down trees and filling public spaces of markets with concrete).

In the context of our study, environmental education, or more broadly education for sustainable development, emerges as a prerequisite for the implementation of processes necessary to meet ‘the needs of the present without compromising the ability of future generations to meet their own needs’ [48]. This concept, formulated several decades ago, has resulted in a number of challenges for the education system. The most crucial ones include a shift from the narrow model of education that promotes the intellect and rational mind to a holistic education that embraces the physical-material, psychological, and spiritual-emotional spheres of human life. Out of concern for our living environment and the global world, it is necessary to ensure access to education for everyone, at all levels, at every stage of human life, and in all social contexts (family, school, work, local community).
